# The Association Between Education and Smoking Prevalence, Independent of Occupation: A Nationally Representative Survey in Japan

**DOI:** 10.2188/jea.JE20180195

**Published:** 2020-03-05

**Authors:** Kimiko Tomioka, Norio Kurumatani, Keigo Saeki

**Affiliations:** Nara Prefectural Health Research Center, Nara Medical University, Nara, Japan

**Keywords:** age difference, education, gender difference, occupation, tobacco smoking

## Abstract

**Background:**

Higher smoking prevalence in less educated persons and manual workers is well known. This study examines the independent relationship of education and occupation with tobacco use.

**Methods:**

We used anonymized data from a nationwide population survey (30,617 men and 33,934 women). Education was divided into junior high school, high school, or university attainment. Occupation was grouped into upper non-manual, lower non-manual, and manual. Poisson regression models stratified by age and gender were used to estimate adjusted prevalence ratio (PR) and 95% confidence interval (CI) for current smoking.

**Results:**

After adjustment for covariates, education, and occupation, education was significantly related to current smoking in both genders; compared to university graduates, PRs of junior high school graduates aged 20–39, 40–64, and ≥65 were 1.74 (95% CI, 1.53–1.98), 1.50 (95% CI, 1.36–1.65), and 1.28 (95% CI, 1.08–1.50) among men, and 3.54 (95% CI, 2.92–4.30), 2.72 (95% CI, 2.29–3.23), and 1.74 (95% CI, 1.14–2.66) among women, respectively. However, significantly higher smoking prevalence in manual than in upper non-manual was found only in men aged 20–64; compared to upper non-manual, the PRs of manual workers aged 20–39, 40–64, and ≥65 were 1.11 (95% CI, 1.02–1.22), 1.18 (95% CI, 1.10–1.27), and 1.10 (95% CI, 0.89–1.37) among men, and 0.95 (95% CI, 0.75–1.20), 0.92 (95% CI, 0.75–1.12), and 0.46 (95% CI, 0.22–0.95) among women, respectively.

**Conclusions:**

Independent of occupation, educational disparities in smoking existed, regardless of age and gender. Occupation-smoking relationship varied with age and gender. Our study suggests that we should pay attention to social inequality in smoking as well as national smoking prevalence.

## INTRODUCTION

The National Cancer Institute and World Health Organization (WHO) have recommended reinforcing tobacco control and eliminating tobacco smoke pollution, because tobacco use is the leading single preventable cause of death worldwide and imposes a heavy economic burden.^1^ According to WHO reports, globally, 14% of all deaths from non-communicable diseases among adults aged 30 years or older are attributable to tobacco (eg, tobacco use is responsible for an estimated 71% of lung cancer deaths and 36% of deaths due to pulmonary problems),^2^ and the estimated economic cost associated with smoking is 1.8% of global gross domestic product.^3^ In Japan, smoking is the most attributable and preventable risk factor for non-communicable diseases^4^^,^^5^ and the population attributable fraction of mortality associated with tobacco smoking is estimated to be 27.8% of all-causes, 38.6% of all cancers, 69.2% of lung cancers, and 60.3% of chronic obstructive pulmonary diseases for men (the corresponding values for women are 6.7%, 5.2%, 19.8%, and 15.6%, respectively).^6^ Therefore, anti-smoking measures are part of a fundamental health promotion plan in all nations, including Japan.

Prior studies indicate that smoking status is associated with numerous factors.^7^^,^^8^ Especially, regarding reports on socioeconomic differences in smoking, many studies have focused on education and occupation, reporting that smoking is more prevalent among persons with less education,^9^^–^^19^ those with lower occupational status,^13^^,^^19^^,^^20^ and among blue-collar workers.^21^^–^^24^ However, prior studies have the following limitations: (1) subjects were limited to working-class participants,^16^^,^^18^^,^^20^^–^^24^ so it is conceivable that prior studies’ findings have healthy worker effects; (2) although there are differences in age and gender groups on academic background, work situation, and smoking habit, stratified analyses by both age and gender were not conducted^9^^,^^11^^,^^13^^,^^17^^–^^20^^,^^22^^,^^23^; and (3) although smoking behavior is affected by many factors,^7^^,^^8^^,^^11^^,^^18^ such as marital status, financial situation, medical conditions, and mental health, prior studies did not have enough variable adjustment.^9^^,^^10^^,^^12^^,^^14^^–^^16^^,^^22^^,^^24^

In Japan, Tabuchi et al investigated smoking inequalities to education^25^; they found differences in age- and gender-specific educational gradients in smoking among Japanese adults, but lacked adequate adjustments and occupational variables. Fukuda et al examined the association between smoking behavior and income considering age, gender, and socioeconomic factors, including employment status, marital status, and residential area, in the Japanese population^26^; they showed gender and age differences in the relation between equivalent household income and smoking, but did not include educational information. It is noted that there is a high correlation between education level and occupation^13^^,^^16^^,^^27^; persons with low educational backgrounds tend to engage in lower-status jobs, while well-educated persons tend to have higher occupational status. Therefore, it is necessary and effective to verify the independent associations of education and occupation with smoking by age and gender groups, with adjustment for important confounding factors, among the general population. In this study, we investigated the cross-sectional relationship between education, occupation, and current smoking of different genders and age groups based on anonymized data from the Comprehensive Survey of Living Conditions (CSLC), which is one of the major national surveys.

## METHODS

### Data

We used anonymized data from the CSLC,^28^ provided by Ministry of Health, Labour and Welfare based on the Statistics Act, Article 36. The CSLC is a nationwide survey that the ministry has held every year since 1986, covering basic matters related to the nation’s everyday living, including health, medicine, pensions, welfare, and income, to obtain basic data required to plan and operate health, welfare, and labor administrations. The CSLC covers households and their membership throughout Japan, and a large-scale survey is implemented every 3 years. In other years, smaller-scale surveys are conducted. In 2010, survey slips were distributed to all households in 5,510 stratified random sampling districts (289,363 households) on June 3, and collected from 229,785 households (response rate, 79.4%). Security measures were implemented to prevent this anonymized data and participants’ personal information from being specified, as prefectural names as well as birthdates and ages were deleted. Regarding age, it was re-coded into 5-year segments, and persons aged 90 and older were re-fined as the oldest group. Additionally, the following households were excluded because they could possibly reveal personal identification: 1) a family size with more than eight persons; 2) single-male-parent households; 3) families who had more than two members in need of nursing care; 4) families who had a large age difference between husband and wife; 5) families who had a small age difference between a parent and child; and 6) families with more than four persons in the same age-group. After these households were excluded, subjects of the anonymized data were randomly selected. Eventually, we were provided with anonymized data from 93,730 people in 36,387 households; the size of resampling corresponding to that of smaller-scale surveys.

### Study participants

The CSLC asked individuals older than 12 years who were not in a hospital/facility a question about smoking status. Additionally, persons aged 19 or younger cannot answer current smoking honestly because of illegality. Therefore, we excluded 29,179 persons from our analyses due to being aged 19 or younger (*n* = 16,951), being an uncertain age (*n* = 111), in a hospital/facility (*n* = 1,587), having uncertain hospitalization/admission (*n* = 1,723), and missing smoking habit data (*n* = 8,807). Eventually, 64,551 persons aged 20 years and older (30,617 men and 33,934 women) were included in this study.

### Measurements

#### Smoking status

The CSLC included the following question: “Do you smoke?”. Response alternatives were “daily smoker”, “occasional smoker” (ie, persons who smoke occasionally but not every day), “ex-smoker” (ie, persons who quitted smoking more than one month previously), or “never-smoker”. Persons whose responses were “daily smoker” and “occasional smoker” were defined as current smokers, and “ex-smoker” and “never-smoker” as non-current smokers.

#### Educational attainment

The first question was “Are you in school or have you finished school?”. Next, persons who had completed their schooling were asked about their educational background, while those who were in school at the time of the survey were asked about current school. We categorized educational attainment as junior high school, high school, and university (including junior college and graduate school) levels of education. In Japan, there is 9-year compulsory education, which corresponds to junior high school graduates. Persons who completed high school have 12 years of education, and those who graduated from university have 16 years of education (14–15 years of education in the case of junior colleges, and 17–22 years of education if graduate school was attended).^29^

#### Occupation

The questionnaire asked all subjects about their working status in May 2010. Those who worked with income were defined as “having a job”. The job classification included twelve kinds of occupations based on the Japan Standard Occupational Classification (JSOC).^30^ In this study, using the data of whether subjects were working or not and the JSOC job classifications, the subjects were classified into upper non-manual (ie, managers and professionals), lower non-manual (ie, clerical, sales, and services workers), manual (ie, manufacturing, transport, machine, construction, mining, protective services, agricultural, forestry, fishery, carrying, cleaning, and packing workers, and others), non-working persons, and those with missing data. These three occupational categories were based on earlier studies.^16^^,^^27^

#### Covariates

According to previous reports in the same field,^7^^,^^8^^,^^18^^,^^31^^,^^32^ the following variables were included as covariates that may correlate with education, occupation, and smoking status: age, housing tenure, family size, marital status, equivalent household expenditure, medical conditions, self-rated health, and psychological distress. Housing tenure was categorized into owner-occupied, privately rented, and socially rented. Family size was categorized into 1 (living alone), 2, 3–4, and ≥5. Marital status was categorized into married, never-married, and widowed/divorced. Monthly equivalent household expenditure (thousand yen) was divided into three categories by tertiles: low (<106), middle (106–160), and high (>160). Medical conditions under treatment, assessed using the multiple-answers-allowed question, were defined as a presence of any of the following health issues: diabetes; obesity; dyslipidemia; thyroid diseases; mental and neurological diseases, including depression, dementia, and Parkinson’s disease; vision disorders; hearing disorders; cardiovascular diseases, including hypertension, stroke, and coronary artery diseases; respiratory diseases, including asthma; diseases of digestive organs, including the stomach, duodenum, liver, and gall bladder; dental diseases; skin disorders, including atopic dermatitis; muscular-skeletal disorders, including gout, rheumatoid arthritis, arthropathy, backache, and osteoporosis; urinary and genital diseases, including renal diseases, prostatic hyperplasia, and menopausal disorders; injuries, including bone fractures; hematological diseases; and cancer. Self-rated health was dichotomized as fine (ie, excellent, very good, or good) versus poor (ie, fair or poor).^32^ Psychological distress was evaluated using the Kessler 6 (range 0–24),^33^ and the presence of psychological distress was defined as a score of ≥5.^34^ A category entitled “missing” was used for values that were missing in responses to questions on the covariates.^35^ Details of the covariates, including the number of missing values according to age, are shown in eTable 1 for men and eTable 2 for women. Additionally, smoking prevalence according to age, gender, and basic characteristics is shown in eTable 3.

### Statistical analysis

To examine age and gender differences in the association between education, occupation, and current smoking, stratified analyses were conducted by age and gender groups; men aged 20–39, men aged 40–64, men aged ≥65, women aged 20–39, women aged 40–64, and women aged ≥65. Poisson regression analyses with a robust variance estimator were carried out using “with” or “without current smoking” as a dependent variable. Independent variables were education and occupation. The results were shown as a prevalence ratio (PR) for current smoking with a 95% confidence interval (CI). Model 1 was adjusted for all covariates. Model 2 was mutually adjusted for education and occupation, in addition to adjustment for all covariates. IBM SPSS Statistics version 24.0 (IBM Corporation, Armonk, NY, USA) was used to perform the statistical analyses, and the null hypothesis was rejected when the probability value was less than 0.05.

### Ethics

In this study, we received approval of use for academic purposes by the Japanese Ministry of Health, Labour and Welfare and were provided data without any information regarding the identification of any individual.

## RESULTS

Characteristics of the participants by age and gender are shown in Table 1. Compared with younger age groups, older age groups had a higher prevalence of persons living in owner-occupied housing, having medical conditions, poor self-rated health, and a junior high school education. Compared to older age groups, younger age groups had a higher prevalence of individuals with psychological distress. People aged 40–64 had the highest prevalence of manual workers and the lowest prevalence of persons with low equivalent household expenditures. These were observed among both genders. The highest proportions of persons living alone were seen in men aged 20–39 and in women aged ≥65.

**Table 1.** Characteristics of analyzed participants by age and gender

	Men (*n* = 30,617)			Women (*n* = 33,934)		
	
	20–39 years	40–64 years	≥65 years	20–39 years	40–64 years	≥65 years
	(*n* = 9,383)	(*n* = 13,847)	(*n* = 7,387)	(*n* = 9,889)	(*n* = 14,715)	(*n* = 9,330)
	*n* (%)	*n* (%)	*n* (%)	*n* (%)	*n* (%)	*n* (%)
Housing tenure: owner-occupied	5,550 (59.1)	10,719 (77.4)	6,424 (87.0)	5,930 (60.0)	11,703 (79.5)	8,003 (85.8)
Family size: one (ie, living alone)	1,236 (13.2)	1,488 (10.7)	765 (10.4)	781 (7.9)	964 (6.6)	2,039 (21.9)
Marital status: married	4,052 (43.2)	11,174 (80.7)	6,218 (84.2)	4,951 (50.1)	11,805 (80.2)	4,767 (51.1)
Equivalent household expenditures: low	3,367 (35.9)	4,063 (29.3)	2,504 (33.9)	3,551 (35.9)	4,180 (28.4)	3,576 (38.3)
Medical conditions under treatment: present	1,469 (15.7)	5,391 (38.9)	5,249 (71.1)	2,154 (21.8)	6,072 (41.3)	6,932 (74.3)
Self-rated health: poor	735 (7.8)	1,732 (12.5)	1,680 (22.7)	993 (10.0)	2,027 (13.8)	2,380 (25.5)
Psychological distress: present	2,770 (29.5)	3,460 (25.0)	1,190 (16.1)	3,274 (33.1)	4,385 (29.8)	2,055 (22.0)
Education: junior high school	445 (4.7)	1,237 (8.9)	2,415 (32.7)	315 (3.2)	1,130 (7.7)	3,739 (40.1)
Occupation: manuals	2,169 (23.1)	3,496 (25.2)	1,006 (13.6)	502 (5.1)	1,507 (10.2)	523 (5.6)

Smoking prevalence according to age, gender, education, and occupation is shown in Table 2. The prevalence of current smokers was 37.7% in men and 11.6% in women, showing a significant gender difference (*P* < 0.001, chi-squared test); regardless of gender, the younger the subjects were, the more frequently they were current smokers (*P* for trend <0.001, Mantel-extension method). For education, among men aged 20–64 and women twenty or older, individuals with a junior high school education had the highest current smoking prevalence. Current smoking prevalence was the lowest in university graduates, regardless of age and gender. Among those aged ≥65, the differences in current smokers across educational categories was diminished. Concerning occupation, among men aged 20–64, manual workers had the highest current smoking prevalence. Among women, the highest prevalence was observed in manual workers aged 20–39, in lower non-manual workers aged 40–64, and in upper non-manual workers aged ≥65.

**Table 2.** Number (and prevalence) of current smoking according to age, gender, education, and occupation

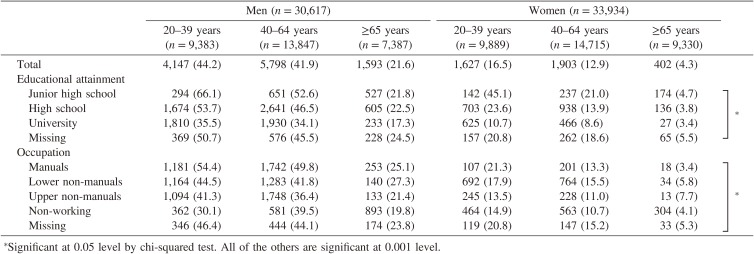

Table 3 shows the adjusted PR for current smoking according to education and occupation, stratified by age and gender. Concerning education, after adjustment for all covariates (model 1), irrespective of gender and age, individuals with a junior high school education were more likely to smoke than those with a university education; these associations were stronger in those aged 20–64 than in those aged ≥65 and in women than in men. Moreover, among men, no matter the age, individuals with a high school education were more likely to smoke compared to persons with a university education. Among women, the association between a high school education and current smoking was observed in those aged 20–64, but not in those aged ≥65. These relationships remained unchanged and significant, even after mutual adjustment for education and occupation (model 2). For junior high school graduates relative to university graduates, among ages 20–39, we found PRs of 1.74 (95% CI, 1.53–1.98) in men and 3.54 (95% CI, 2.92–4.30) in women; among ages 40–64, PRs were 1.50 (95% CI, 1.36–1.65) in men and 2.72 (95% CI, 2.29–3.23) in women; and among ages ≥65, PRs were 1.28 (95% CI, 1.08–1.50) in men and 1.74 (95% CI, 1.14–2.66) in women. For high school graduates relative to university graduates, among ages 20–39, we found PRs of 1.42 (95% CI, 1.32–1.53) in men and 2.09 (95% CI, 1.86–2.34) in women; among ages 40–64, PRs were 1.31 (95% CI, 1.24–1.40) in men and 1.67 (95% CI, 1.49–1.88) in women; and among ages ≥65, PRs were 1.24 (95% CI, 1.06–1.45) in men and 1.22 (95% CI, 0.80–1.85) in women.

**Table 3.** Adjusted prevalence ratio for current smoking according to education and occupation, stratified by age and gender

	Model 1: Adjusted for covariates			Model 2: Mutually adjusted for education and occupation		
	
	20–39 years	40–64 years	≥65 years	20–39 years	40–64 years	≥65 years
	PR^a^ (95% CI)	PR^a^ (95% CI)	PR^a^ (95% CI)	PR^b^ (95% CI)	PR^b^ (95% CI)	PR^b^ (95% CI)
**Men**						
Education (reference: university)						
High school	1.46 (1.37–1.57)^*^	1.36 (1.29–1.45)^*^	1.25 (1.08–1.46)^*^	1.42 (1.32–1.53)^*^	1.31 (1.24–1.40)^*^	1.24 (1.06–1.45)^*^
Junior high school	1.77 (1.56–2.00)^*^	1.58 (1.44–1.74)^*^	1.29 (1.10–1.52)^*^	1.74 (1.53–1.98)^*^	1.50 (1.36–1.65)^*^	1.28 (1.08–1.50)^*^
Missing	1.37 (1.22–1.53)^*^	1.33 (1.21–1.47)^*^	1.34 (1.11–1.61)^*^	1.35 (1.20–1.52)^*^	1.29 (1.17–1.42)^*^	1.33 (1.09–1.63)^*^
Occupation (reference: upper non-manuals)						
Lower non-manuals	1.09 (1.01–1.19)^*^	1.13 (1.05–1.21)^*^	1.24 (0.98–1.58)^†^	1.08 (0.99–1.17)^†^	1.09 (1.02–1.18)^*^	1.20 (0.95–1.53)
Manuals	1.29 (1.19–1.41)^*^	1.32 (1.24–1.42)^*^	1.17 (0.95–1.45)	1.11 (1.02–1.22)^*^	1.18 (1.10–1.27)^*^	1.10 (0.89–1.37)
Non-working	0.82 (0.72–0.93)^*^	1.12 (1.01–1.24)^*^	1.09 (0.91–1.32)	0.79 (0.69–0.89)^*^	1.05 (0.94–1.16)	1.06 (0.88–1.28)
Missing	1.12 (0.99–1.27)^†^	1.19 (1.07–1.32)^*^	1.15 (0.91–1.45)	1.03 (0.91–1.18)	1.11 (0.99–1.23)^†^	1.06 (0.83–1.34)
**Women**						
Education (reference: university)						
High school	2.09 (1.87–2.33)^*^	1.68 (1.50–1.89)^*^	1.17 (0.77–1.77)	2.09 (1.86–2.34)^*^	1.67 (1.49–1.88)^*^	1.22 (0.80–1.85)
Junior high school	3.49 (2.88–4.21)^*^	2.66 (2.25–3.14)^*^	1.65 (1.09–2.50)^*^	3.54 (2.92–4.30)^*^	2.72 (2.29–3.23)^*^	1.74 (1.14–2.66)^*^
Missing	1.85 (1.55–2.21)^*^	2.09 (1.79–2.44)^*^	1.46 (0.92–2.31)	1.84 (1.54–2.20)^*^	2.11 (1.80–2.47)^*^	1.52 (0.95–2.45)^†^
Occupation (reference: upper non-manuals)						
Lower non-manuals	1.30 (1.12–1.51)^*^	1.32 (1.14–1.53)^*^	0.66 (0.35–1.26)	1.07 (0.92–1.24)	1.12 (0.96–1.31)	0.63 (0.33–1.20)
Manuals	1.44 (1.14–1.81)^*^	1.21 (0.99–1.46)^†^	0.53 (0.26–1.09)^†^	0.95 (0.75–1.20)	0.92 (0.75–1.12)	0.46 (0.22–0.95)^*^
Non-working	1.09 (0.92–1.28)	1.06 (0.90–1.24)	0.72 (0.41–1.26)	0.88 (0.74–1.03)	0.89 (0.75–1.04)	0.64 (0.36–1.14)
Missing	1.49 (1.20–1.86)^*^	1.27 (1.03–1.56)^*^	0.70 (0.37–1.35)	1.10 (0.88–1.38)	1.00 (0.81–1.24)	0.61 (0.31–1.19)

CI, confidence interval; PR, prevalence ratio.

^*^P < 0.05, ^†^*P* < 0.10.

^a^Adjusted for age, housing tenure, family size, marital status, equivalent household expenditures, medical conditions, self-rated health, and psychological distress.

^b^In addition to the factors in Model 1, education and occupation were included (ie, Model 2 was mutually adjusted for education and occupation).

In terms of occupation, among men, after adjustment for all covariates (model 1), manual and lower non-manual workers were more likely to be current smokers compared to upper non-manual workers in ages 20–64. After mutual adjustment for education and occupation (model 2), these relationships were attenuated, but the following associations remained significant: compared to upper non-manual workers, the PRs for current smoking were significantly higher among manual workers aged 20–64 (PR 1.11; 95% CI, 1.02–1.22 in ages 20–39 and 1.18; 1.10–1.27 in ages 40–64), and among lower non-manual workers aged 40–64 (PR 1.09; 95% CI, 1.02–1.18). Among women, although manual workers aged 20–39 and lower non-manual workers aged 20–64 were more likely to smoke compared to upper non-manual workers in the adjusted model for covariates (model 1), these significant differences were not found in the mutually adjusted model for education and occupation (model 2). Among women aged ≥65, after mutual adjustment for education and occupation (model 2), manual workers were less likely to be current smokers than upper non-manual workers: PR 0.46 (95% CI, 0.22–0.95) in female manual workers over the age of 65.

## DISCUSSION

In this study, we revealed that, independent of occupation, educational attainment was significantly related to current smoking among the Japanese general population and that educational disparities in smoking were larger among the younger generation than among older people, especially among women aged 20–39. As for occupation, among men and women of working age, manual and lower non-manual workers were more prone to currently smoke than upper non-manual workers, but additional adjustments for education weakened the association between occupation and smoking; among men aged 20–64, significant differences in smoking prevalence between manual and upper non-manual workers remained, while among women aged 20–64, occupation was not associated with current smoking.

Generally, education is established early in life, and tends to be stable. In contrast, occupation is determined comparatively late in life, and can be changed during midlife for better or for worse. Smokers often begin tobacco use in early adulthood, and initiate quitting after middle age. Therefore, it is thought that education has a profound effect on smoking initiation on the younger population, while occupation can affect smoking cessation of the middle-age and older population.^19^^,^^36^ With respect to the association between education and smoking, less educated persons are less likely to have the opportunity to acquire knowledge about smoking and its health effects than those having higher education, leading to a higher prevalence of current smoking in those individuals.^37^ Additionally, living in a society that demands high academic qualifications seems to be one reason for increased difference in smoking prevalence between university-educated persons and junior high school graduates among young people. In Japan, the percentage of students enrolling in universities and junior colleges was reported to be 14.9% in 1960, 41.3% in 1980, 49.4% in 2000, and 57.7% in 2010 among men; in women those same years, the totals were 5.5%, 33.3%, 48.7%, and 56.0%, respectively.^38^ The prevalence of higher education in recent years leads to a domination by highly educated people, meaning those who had only graduated from junior high school may be more likely to be at a disadvantage in employment and marriage, provoking a widening gap between high-education haves and have-nots in the younger generation.^39^ Yet another reason why inequality in smoking prevalence is smaller in older people, is that Japanese smoking prevalence in the 1970s, when currently older adults were one of the younger generations, was reported to be about 80% in men^40^; regardless of socioeconomic status, smoking used to be prevalent among Japanese men. According to a model of smoking epidemic patterns in society,^41^ with a decline in smoking prevalence, tobacco use is more frequent among the low socioeconomic group than among the affluent, and a decline in female prevalence typically lags behind that of men by several decades. Our results showing smoking prevalence to be inversely associated with educational level, particularly evident in young women, agree with the findings of previous research^10^^,^^14^ and smoking epidemic patterns based on theories.^41^

For the association between occupation and smoking, many studies have reported a significant association between lower level occupations and a higher percentage of current smokers.^13^^,^^19^^–^^24^ It is well known that people with lower level occupations have more difficulties in successfully quitting smoking than those with higher level occupations because they are less likely to receive treatment and support to quit.^42^ Another reason for a high rate of smoking in manual workers may be their hard physical tasks or a common practice of smoking at their workplace.^21^^,^^43^ However, in this study, after mutual adjustment for education, only men aged 20–64 had a finding consistent with previous studies: manual workers were more likely to be current smokers than upper non-manual workers. For women, among those aged ≥65, upper non-manual workers had a significantly higher current smoking prevalence than manual workers. Our results may be supported by a prior Japanese study that reported having a responsible position can bring about a positive emotional effect for older men but creates a psychological burden for older women.^44^

Most smokers cite stress relief as a perceived benefit and motive for smoking.^45^ However, because nicotine withdrawal symptoms can induce psychological discomfort, such as irritability, anxiety, and depression,^46^ smokers may misunderstand relief of nicotine withdrawal symptoms as stress relief; a prior study based on systematic review and meta-analysis has confirmed that smoking cessation can reduce stress and improve mental health.^47^ Because the motive “Smoking helps me cope with stress” is reported to be associated with the female gender, younger age, and decreasing social grade,^45^ educating that smoking never has beneficial effects on mental health, including stress relief, and instructing the proper method to mitigate nicotine withdrawal symptoms may be a key to the smoking-cessation support for young women with low education.

This study’s strengths are as follows: 1) our findings are based on nationally representative data, and analyzed subjects include residents without occupations, which can avoid the healthy workers effect and has an advantage in generalization; 2) this study has sufficient covariates, including other socioeconomic status, such as housing tenure, family size, marital status, and equivalent household expenditures, and health conditions, such as medical conditions, self-rated health, and psychological distress (ie, we adjusted for possible confounding factors); and 3) we used data from a large government study, which permits performance of stratified analyses according to age and gender.

We have several limitations. First, since all information used in this study relies on respondents’ self-reporting, we have no objective data. Previous studies suggest that people are reluctant to disclose socially undesirable behavior because of a penchant for giving a good account of oneself.^48^ Additionally, people with higher education tend to be more secretive about socially undesirable behavior than the less educated.^49^ Therefore, well-educated smokers may report false answers regarding smoking, compared to smokers with lower academic backgrounds. This can produce differential misclassification and may overestimate the association between lower educational level and current smoking. Second, some researchers suggest that women are more affected by their husbands’ and householders’ occupation rather than their own.^13^^,^^27^ Additionally, in our study, women make up a large number of unemployed persons and a small number of upper non-manuals and manual workers compared to men. This might create insufficient statistical power in evaluating the association between occupation and smoking among women. Third, the CSLC asked participants about the previous month’s employment but did not about their work history. Previous studies of older adults suggest that midlife occupational activities have a greater effect on old-age health than working status in later life.^50^^,^^51^ Therefore, the association between education, occupation, and current smoking among older people needs to be confirmed in further studies based on work experience from early adulthood through old age. Fourth, the JSOC, which is consistent with the International Standard Classification of Occupations established by the International Labour Organization and has been commonly used not only in national statistical surveys^52^ but also in epidemiology research,^53^ was adapted as an assessment of occupation by the CSLC. However, a prior study suggests that it is difficult to capture occupation-related health inequalities in Japan solely through the JSOC.^54^ This concern may lead to weakening the association between occupation and smoking in our study. Finally, a handful of people with potential personal identification data, such as single-male-parent households, families who have several members in need of nursing care, and families who have age differences departing from what is socially acceptable, were eliminated from the anonymized data. Because ineligible people for the anonymized data are considered to be part of the socioeconomically disadvantaged population, anonymized data may be distorted towards the more advantaged. Therefore, our findings may underestimate the association between SES factors and smoking, compared to those from the full dataset.

### Conclusions

Independent of other socioeconomic status and health conditions, both men and women have educational inequalities in smoking, particularly among young women, while the association between occupation and smoking varies with age and gender. The Japanese government has launched a national movement for health promotion aimed at extending healthy life expectancy and reducing health disparities; reducing the rates of smoking among adults is one of the important targets.^55^ Our findings indicate that, when it comes to smoking, we should pay attention not only to national smoking prevalence but also to smoking prevalences of specific populations and social inequalities.

## APPENDIX A. SUPPLEMENTARY DATA

eTable 1. Characteristics of the 30,617 male subjects

eTable 2. Characteristics of the 33,934 female subjects

eTable 3. Number (and prevalence) of current smoking according to age, gender, and basic characteristics
