# Supplementary material for: The Association Between Education and Smoking Prevalence, Independent of Occupation: A Nationally Representative Survey in Japan
Source: J Epidemiol. 2020 Mar 5;30(3):136–42. doi: 10.2188/jea.JE20180195 (PMC7025916; doi:10.2188/jea.JE20180195)
Supplement: Supplementary file 1 [file je-30-136-s001.pdf]

eTable 1. Characteristics of the 30,617 male subjects

		20–39 years (n=9,383) n (%)	40–64 years (n=13,847) n (%)	≥65 years (n=7,387) n (%)
Housing tenure	Owner-occupied	5,550 (59.1)	10,719 (77.4)	6,424 (87.0)
	Privately rented	2,366 (25.2)	1,718 (12.4)	446 (6.0)
	Socially rented	1,467 (15.6)	1,410 (10.2)	517 (7.0)
Family size	≥5	1,649 (17.6)	2,302 (16.6)	834 (11.3)
	3–4	5,239 (55.8)	7,041 (50.8)	2,186 (29.6)
	2	1,259 (13.4)	3,016 (21.8)	3,602 (48.8)
	1: living alone	1,236 (13.2)	1,488 (10.7)	765 (10.4)
Marital status	Married	4,052 (43.2)	11,174 (80.7)	6,218 (84.2)
	Never-married	5,202 (55.4)	1,866 (13.5)	158 (2.1)
	Widowed/divorced	129 (1.4)	807 (5.8)	1,011 (13.7)
Monthly equivalent household expenditures	Low: lower tertile	3,367 (35.9)	4,063 (29.3)	2,504 (33.9)
	Middle: middle tertile	2,986 (31.8)	4,142 (29.9)	2,148 (29.1)
	High: upper tertile	2,562 (27.3)	4,895 (35.4)	2,342 (31.7)
	Missing	468 (5.0)	747 (5.4)	393 (5.3)
Medical conditions under treatment	Absent	7,705 (82.1)	8,268 (59.7)	2,048 (27.7)
	Present	1,469 (15.7)	5,391 (38.9)	5,249 (71.1)
	Missing	209 (2.2)	188 (1.4)	90 (1.2)
Self-rated health	Very good/good/fair	8,283 (88.3)	11,532 (83.3)	5,033 (68.1)
	Poor/very poor	735 (7.8)	1,732 (12.5)	1,680 (22.7)
	Missing	365 (3.9)	583 (4.2)	674 (9.1)
Psychological distress based on the K6 score	Absent: K6 score ≤4	6,260 (66.7)	9,535 (68.9)	4,722 (63.9)
	Present: K6 score ≥5	2,770 (29.5)	3,460 (25.0)	1,190 (16.1)
	Missing	353 (3.8)	852 (6.2)	1,475 (20.0)
Educational attainment	Junior high school	445 (4.7)	1,237 (8.9)	2,415 (32.7)
	High school	3,115 (33.2)	5,682 (41.0)	2,691 (36.4)
	University	5,095 (54.3)	5,661 (40.9)	1,350 (18.3)
	Missing	728 (7.8)	1,267 (9.1)	931 (12.6)
Occupation	Manual[a]	2,169 (23.1)	3,496 (25.2)	1,006 (13.6)
	Lower non-manual[b]	2,618 (27.9)	3,073 (22.2)	513 (6.9)
	Upper non-manual[c]	2,646 (28.2)	4,800 (34.7)	621 (8.4)
	Non-working	1,204 (12.8)	1,471 (10.6)	4,516 (61.1)
	Missing	746 (8.0)	1,007 (7.3)	731 (9.9)

[a]Manufacturing, transport, machine, construction, mining, protective services, agricultural, forestry, fishery, carrying, cleaning, and packing workers, and others.

[b]Clerical, sales, and services workers.

[c]Managers and professionals.

eTable 2. Characteristics of the 33,934 female subjects

		20–39 years (n=9,889) n (%)	40–64 years (n=14,715) n (%)	≥65 years (n=9,330) n (%)
Housing tenure	Owner-occupied	5,930 (60.0)	11,703 (79.5)	8,003 (85.8)
	Privately rented	2,410 (24.4)	1,618 (11.0)	597 (6.4)
	Socially rented	1,549 (15.7)	1,394 (9.5)	730 (7.8)
Family size	≥5	1,905 (19.3)	2,288 (15.5)	1,169 (12.5)
	3–4	5,774 (58.4)	7,616 (51.8)	2,609 (28.0)
	2	1,429 (14.5)	3,847 (26.1)	3,513 (37.7)
	1: living alone	781 (7.9)	964 (6.6)	2,039 (21.9)
Marital status	Married	4,951 (50.1)	11,805 (80.2)	4,767 (51.1)
	Never-married	4,502 (45.5)	1,144 (7.8)	244 (2.6)
	Widowed/divorced	436 (4.4)	1,766 (12.0)	4,319 (46.3)
Monthly equivalent household expenditures	Low: lower tertile	3,551 (35.9)	4,180 (28.4)	3,576 (38.3)
	Middle: middle tertile	3,147 (31.8)	4,406 (29.9)	2,702 (29.0)
	High: upper tertile	2,676 (27.1)	5,352 (36.4)	2,543 (27.3)
	Missing	515 (5.2)	777 (5.3)	509 (5.5)
Medical conditions under treatment	Absent	7,530 (76.1)	8,445 (57.4)	2,264 (24.3)
	Present	2,154 (21.8)	6,072 (41.3)	6,932 (74.3)
	Missing	205 (2.1)	198 (1.3)	134 (1.4)
Self-rated health	Very good/good/fair	8,623 (87.2)	12,035 (81.8)	6,162 (66.0)
	Poor/very poor	993 (10.0)	2,027 (13.8)	2,380 (25.5)
	Missing	273 (2.8)	653 (4.4)	788 (8.4)
Psychological distress based on the K6 score	Absent: K6 score ≤4	6,320 (63.9)	9,398 (63.9)	5,354 (57.4)
	Present: K6 score ≥5	3,274 (33.1)	4,385 (29.8)	2,055 (22.0)
	Missing	295 (3.0)	932 (6.3)	1,921 (20.6)
Educational attainment	Junior high school	315 (3.2)	1,130 (7.7)	3,739 (40.1)
	High school	2,979 (30.1)	6,770 (46.0)	3,602 (38.6)
	University	5,839 (59.0)	5,407 (36.7)	802 (8.6)
	Missing	756 (7.6)	1,408 (9.6)	1,187 (12.7)
Occupation	Manual[a]	502 (5.1)	1,507 (10.2)	523 (5.6)
	Lower non-manual[b]	3,874 (39.2)	4,922 (33.4)	591 (6.3)
	Upper non-manual[c]	1,818 (18.4)	2,066 (14.0)	168 (1.8)
	Non-working	3,124 (31.6)	5,254 (35.7)	7,421 (79.5)
	Missing	571 (5.8)	966 (6.6)	627 (6.7)

[a]Manufacturing, transport, machine, construction, mining, protective services, agricultural, forestry, fishery, carrying, cleaning, and packing workers, and others.

[b]Clerical, sales, and services workers.

[c]Managers and professionals.

eTable 3. Number (and prevalence) of current smoking according to age, gender, and basic characteristics

	Men (n=30,617)			Women (n=33,934)		
	20–39 years (n=9,383)	40–64 years (n=13,847)	≥65 years (n=7,387)	20–39 years (n=9,889)	40–64 years (n=14,715)	≥65 years (n=9,330)
Housing tenure						
Owner-occupied	2,482 (44.7)	4,308 (40.2)	1,284 (20.0)	858 (14.5)	1,233 (10.5)	263 (3.3)
Privately rented	1,025 (43.3)	844 (49.1)	170 (38.1)	447 (18.5)	393 (24.3)	77 (12.9)
Socially rented	640 (43.6)	646 (45.8)	139 (26.9)	322 (20.8)	277 (19.9)	62 (8.5)
Family size						
≥5	798 (48.4)	1,051 (45.7)	153 (18.3)	341 (17.9)	264 (11.5)	38 (3.3)
3–4	2,356 (45.0)	2,859 (40.6)	492 (22.5)	888 (15.4)	907 (11.9)	104 (4.0)
2	538 (42.7)	1,182 (39.2)	712 (19.8)	253 (17.7)	525 (13.6)	130 (3.7)
1: living alone	455 (36.8)	706 (47.4)	236 (30.8)	145 (18.6)	207 (21.5)	130 (6.4)
Marital status						
Married	1,996 (49.3)	4,509 (40.4)	1,280 (20.6)	786 (15.9)	1,302 (11.0)	175 (3.7)
Never-married	2,053 (39.5)	844 (45.2)	61 (38.6)	658 (14.6)	175 (15.3)	27 (11.1)
Widowed/divorced	98 (76.0)	445 (55.1)	252 (24.9)	183 (42.0)	426 (24.1)	200 (4.6)
Monthly equivalent household expenditures						
Low	1,553 (46.1)	1,825 (44.9)	569 (22.7)	651 (18.3)	616 (14.7)	151 (4.2)
Middle	1,289 (43.2)	1,751 (42.3)	460 (21.4)	508 (16.1)	579 (13.1)	118 (4.4)
High	1,095 (42.7)	1,891 (38.6)	471 (20.1)	364 (13.6)	579 (10.8)	96 (3.8)
Missing	210 (44.9)	331 (44.3)	93 (23.7)	104 (20.2)	129 (16.6)	37 (7.3)
Medical conditions under treatment						
Absent	3,500 (45.4)	3,777 (45.7)	629 (30.7)	1,215 (16.1)	1,147 (13.6)	132 (5.8)
Present	560 (38.1)	1,933 (35.9)	943 (18.0)	372 (17.3)	730 (12.0)	262 (3.8)
Missing	87 (41.6)	88 (46.8)	21 (23.3)	40 (19.5)	26 (13.1)	8 (6.0)
Self-rated health (SRH)[a]						
Good	3,665 (44.2)	4,830 (41.9)	1,116 (22.2)	1,359 (15.8)	1,455 (12.1)	252 (4.1)
Poor	325 (44.2)	701 (40.5)	333 (19.8)	223 (22.5)	348 (17.2)	105 (4.4)
Missing	157 (43.0)	267 (45.8)	144 (21.4)	45 (16.5)	100 (15.3)	45 (5.7)
Psychological distress based on the K6 score						
Absent	2,754 (44.0)	3,935 (41.3)	999 (21.2)	922 (14.6)	1,050 (11.2)	221 (4.1)
Present	1,223 (44.2)	1,493 (43.2)	271 (22.8)	646 (19.7)	711 (16.2)	92 (4.5)
Missing	170 (48.2)	370 (43.4)	323 (21.9)	59 (20.0)	142 (15.2)	89 (4.6)

[a] Subjects who reported very good, good, or fair were defined as good SRH, while those who reported poor or very poor were defined as poor SRH.
